# Rapid, simple, and simultaneous electrochemical determination of cadmium, copper, and lead in Baijiu using a novel covalent organic framework based nanocomposite

**DOI:** 10.3389/fchem.2024.1374898

**Published:** 2024-03-07

**Authors:** Liangyun Yu, Jingjing Zhang, Jiajun Li, Liangju Sun, Qi Zhang, Bairen Yang, Mingquan Huang, Baocai Xu

**Affiliations:** ^1^ School of Light Industry, Beijing Technology and Business University, Beijing, China; ^2^ School of Environmental Science and Engineering, Yancheng Institute of Technology, Yancheng, China

**Keywords:** Baijiu, covalent organic frameworks (COFs), differential pulsed anodic stripping voltammetry (DPASV), heavy metal ions, graphene

## Abstract

It is of great significance to develop a simple and rapid electrochemical sensor for simultaneous determination of heavy metal ions (HMIs) in Baijiu by using new nanomaterials. Here, graphene (GR) was utilized to combine with covalent organic frameworks (COFs) that was synthesized via the aldehyde-amine condensation between 2, 5-dimethoxyterephthalaldehyde (DMTP) and 1, 3, 5-tris(4-aminophenyl) benzene (TAPB) to prepare a new GR/COF_DPTB_/GCE sensor for electrochemical sensing multiple HMIs. Compared with the glass carbon electrode (GCE), GR/GCE and COF_DPTB_/GCE, the developed sensor exhibited excellent electrochemical analysis ability for the simultaneous detection of Cd^2+^, Pb^2+^, and Cu^2+^ owing to the synergistically increased the specific surface area, the periodic porous network and plenty of effective binding sites, as well as the enhanced conductivity. Under the optimized experimental parameters, the proposed sensor showed good linearity range of 0.1–25 μM for Cd^2+^, and both 0.1–11 μM for Pb^2+^ and Cu^2+^ with the detection limits of Cd^2+^, Pb^2+^, and Cu^2+^ being 0.011 μM, 8.747 nM, and 6.373 nM, respectively. Besides, the designed sensor was successfully applied to the simultaneous detection of the three HMIs in Baijiu samples, suggesting its good practical application performance and a new method for the rapid detection of HMIs being expended.

## 1 Introduction

Chinese liquor (Baijiu) is the national liquor of China and one of the six distilled spirits in the world, together with brandy, whiskey, vodka, rum and gin (Liu and Sun, 2018; Song et al., 2021; Tu et al., 2022). The material composition of Baijiu has its own feature, which form the characteristics of complex composition and changeable taste. Baijiu not only has great varieties, different flavor types, but also has different brewing techniques. In the process of Baijiu brewing and storage, heavy metal ions (HMIs) are easy to be introduced from the raw materials and utensils used for processing (Song et al., 2018; Wang X. X. et al., 2020; Huang et al., 2020). HMIs in Baijiu have dual functions: On the one hand, they directly control the flavor of liquor. HMIs can gradually dissolve from the clay pot during the aging period of liquor, and these metal cations will combine with oxyethyl anion to form colloidal particles with large specific surface area and strong absorptivity, which dominate the uniform distribution of alcohols, acids, esters, aldehydes, ketones and other trace aroma compounds in Baijiu body, resulting in the pleasant and harmonious taste of aged liquor (Jiang et al., 2019). On the other hand, excessive intake of them through liquor will harm human health (Iwegbue et al., 2014; Zheng et al., 2021). For example, Cd^2+^ can lead to renal dysfunction, osteoporosis, metabolic disorders and cancer (Wu et al., 2020). Excessive intake of Pb^2+^ can lead to anemia, mental decline, kidney and liver damage (Qi et al., 2022). Although Cu^2+^ is an essential element for the human body, excessive Cu^2+^ can also cause liver, gastrointestinal and kidney damage (Zhang et al., 2020). Moderate drinking is healthy and essential to a better life, as it has been reported to help reduce cardiovascular disease-related risk factors such as high-density lipoprotein (HDL) cholesterol level and high blood pressure (Ren et al., 2021). Therefore, it is of great significance to develop an efficient and convenient method for determination of trace HMIs in Baijiu.

The methods commonly used for HMIs detection in Baijiu include inductively coupled plasma mass spectrometry (ICP-MS) (Song et al., 2018; Zhang et al., 2019; Huang et al., 2020), flame atomic absorption spectrometry (FAAS) (Zhu, 2016) and graphite furnace atomic absorption spectrometry (GFAAS) (Lang et al., 2019). Although these methods have high detection sensitivity and can simultaneously detect a variety of HMIs, they are high testing cost, expensive and cumbersome operation. By contrast, the electrochemical method not only has the advantages of traditional detection methods, but also has the characteristics of low detection limit, fast response speed, high selectivity, simple operation and field detection, which is worthy of popularization and application (Han et al., 2020).

Designing new working electrode materials or developing new methods to modify the working electrode is the key problem to improve the performance of electrochemical sensors for the HMIs detection (Zhou et al., 2017). In recent years, various nanomaterials have been widely used as electrode modifiers in the preparation of electrochemical sensors because of their large specific surface area, abundant active sites and high adsorption capacity for HMIs (Karimian et al., 2019; Yu et al., 2020). Covalent organic frameworks (COFs), connected by organic ligands through strong covalent bonds, are periodic stacked porous crystalline polymers composed of C, H, O, N, B and other light elements (Tan et al., 2021). COFs play a critical role in the catalysis, sensing, energy storage and materials science based on their high specific surface, designability, easily functionalized porous structure and highly ordered properties (Tan et al., 2019). However, the poor conductivity of COFs limits their application in the field of the electrochemical sensors (Zhang et al., 2018b; Sun et al., 2019a; Ma et al., 2019). In recent years, many COFs-based composites have been developed, such as COFs combined with amino-functionalized multi-walled carbon nanotubes (Sun et al., 2017), molybdenum disulfide (Sun et al., 2019b), graphene oxide (Sun et al., 2019a), amine functionalized reduced graphene oxide (Wang et al., 2015), carbon nanotubes (Xu F. et al., 2015; Zhu et al., 2021), gold nanoparticles (Zhu et al., 2020) and C_60_ (Yuan et al., 2022). The electrochemical sensors based on the COFs composites show better sensing performance than those based on pure COFs.

**FIGURE 1:**
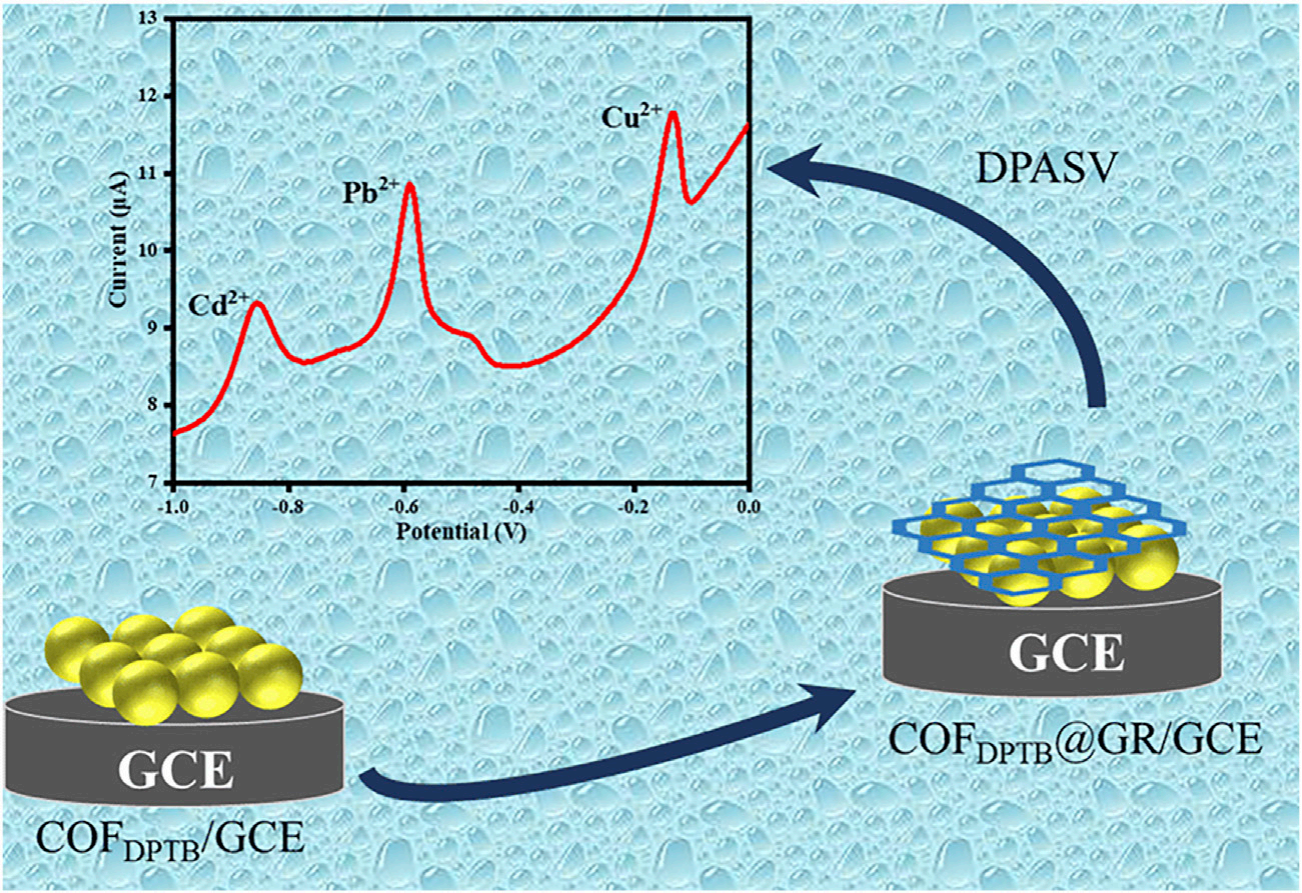
The construction principle and process of the GR/COF_DPTB_/GCE sensor.

Graphene (GR) is a two-dimensional nanomaterial that has high specific surface area, easy functionalization, good mechanical stability and excellent electrical conductivity (Zhong et al., 2018; Baig et al., 2019). Inspired by this, a novel two-dimensional COF_DPTB_ was synthesized via the aldehyde-amine Schiff-base condensation reaction of 1,3, 5-tri (4-aminophenyl) benzene (TAPB) and 2, 5-dimethoxy-p-phenyldiformaldehyde (DMTP), which has a large specific surface area and exhibits good stability against strong acids and bases (Xu H. et al., 2015). Then, it was combined with GR to modify the glass carbon electrode (GCE) layer by layer to construct GR/COF_DPTB_/GCE. The sensor had good differential pulsed anodic stripping voltammetry (DPASV) response to trace of Cd^2+^, Pb^2+^, and Cu^2+^. In this study, experimental parameters such as the dosage ratio of modified material, the buffer pH, the deposition potential and deposition time were optimized. And the selectivity, the stability, the reproducibility, the detection limit and other electrochemical properties of the platform were also discussed. Figure 1 illustrates the construction principle and process of the GR/COF_DPTB_/GCE sensor.

## 2 Experimental section

### 2.1 Reagents and materials

2, 5-dimethoxyterephthalaldehyde (DMTP) was purchased from Alfa Aesar Chemical Co., Ltd. (Shanghai, China). Graphene (GR), 1, 3, 5-tris(4-aminophenyl) benzene (TAPB), sodium phosphate dibasic dodecahydrate (Na_2_HPO_4_·12H_2_O), formic acid and n-butanol were bought from Shanghai Macklin Biochemical Co., Ltd. (China). 1, 4-dioxane and anhydrous sodium dihydrogen phosphate (NaH_2_PO_4_) were purchased from Aladdin Industrial Co., Ltd. (Shanghai, China). Acetone and lactic acid were supplied by Sinopharm Chemical Reagent Co., Ltd. (Shanghai, China). Methanol, acetic acid and tetrahydrofuran (THF) were provided from Tianjin Fuchen Chemical Reagent Co., Ltd. (China). Ethanol was obtained from Mairuida Technology Co., Ltd. (Beijing, China). Ultrapure water (18.25 MΩ·cm) was prepared by Jingjiang Hengxin Environmental Protection Equipment Co., Ltd. (Jiangsu, China). Phosphate buffers (PBS, 0.1 M) at different pH were achieved by mixing different proportions of Na_2_HPO_4_ and Na_2_HPO_4_·12H_2_O. All chemicals were of analytical grade quality and were used without further purification.

### 2.2 Apparatus

Fourier-transform infrared spectroscopy (FTIR) measurements were recorded on a Fourier-transform infrared spectrometer (Nicolet iS10, United States). The X-ray diffraction (XRD) spectra were recorded on an X'Pert3 Powder multifunctional X-ray diffractometer (PANalytical, Holland) in the range of 2θ = 0.5°–80° with Cu-Kα radiation (λ = 1.5418 Å). Scanning electron microscope (SEM) images were obtained on a Zeiss Supra 55 field emission scanning electron microscope. A JEM-2100 transmission electron microscope (Japan) was used to obtain the transmission electron microscopy (TEM) images. High-resolution transmission electron microscopy (HRTEM) and energy dispersive X-ray (EDX) spectrum studies of COF_DPTB_ were carried out on a Tecnai-G2 F30 S-TWIN microscope (Philips, Netherlands). A V-Sorb 2800 (Gold APP Instruments Corporation China) analyzer was used to collect the Brunauer-Emmett-Teller (BET) surface area and pore volume of COF_DPTB_. The all electrochemical experiments were performed on a CHI760 electrochemical workstation (Shanghai Chenhua Instrument, China) with a three-electrode system containing a calomel reference electrode in the saturated KCl solution at room temperature, a platinum wire counter electrode and a GR/COF_DPTB_/GCE (3 mm diameter) as the working electrode.

### 2.3 Preparation of various electrodes

COF_DPTB_ was prepared by the aldehyde-amine Schiff-base condensation reaction of TAPB and DMTP as our previous work (Yu et al., 2023). Specially, 85.0 mg DMTP and 105.0 mg TAPB were firstly dissolved in 45.0 mL mixed solution of 1, 4-dioxane, n-butanol and methanol (volume ratio of 4:4:1) for 30 min by ultrasound. Secondly, 0.5 mL of 3.0 M acetic acid was added by drops and the obtained solution was reacted at room temperature for 2 h. Then, another 4.5 mL of 3.0 M acetic acid was added drop by drop and the mixed solution was placed in an oven at 70°C for 24 h. Next, the solution was cooled naturally to room temperature and the solid product was collected by centrifugation and washed three times with acetone and THF, respectively. The yellow powder of COF_DPTB_ was obtained by vacuum drying at 50°C for 24 h. Before each modification, the GCE was ground on the polishing pad with 0.05 μm Al_2_O_3_ powder until the electrode surface was as smooth as a mirror. Then it was washed ultrasonically with ethanol and water for 3 min in turn and dried. 1.0 mg COF_DPTB_ and 1.0 mg GR were separately dispersed in 1.0 mL H_2_O and treated by ultrasound for about 1 h. The COF_DPTB_/GCE and GR/GCE was obtained by casting 10 μL of COF_DPTB_ suspension and 10 μL of GR suspension on the surface of the pretreated GCE and then dried under the infrared light, respectively. The GR/COF_DPTB_/GCE was prepared layer by layer by casting 10 μL of GR suspension on the surface of COF_DPTB_/GCE and then dried under the infrared light. Lastly, 5.0 μL of 0.5% Nafion was dropped on the modified electrode surfaces and dried for use.

### 2.4 Electrochemical detection of HIMs

DPASV was employed for the high sensitivity detection of Cd^2+^, Pb^2+^, and Cu^2+^ in 10 mL of PBS solution (0.1 M, pH 4.0) under nitrogen atmosphere. DPASV was performed at the potential range from −1.2–0 V (vs. SCE) with the pulse amplitude of 50 mV, the pulse width of 0.2 s, the potential increment of 5 mV, and the quiet time of 10 s. Before every DPASV measurement, nitrogen was firstly injected for 30 min, and then an electrochemical deposition step was carried out at −1.2 V for 260 s and left for 10 s. After each test, a 300 s cleaning step was followed under agitation conditions to remove the residual HMIs at 0.2 V. The same experimental condition was applied for the individual detection as well as the simultaneous one of HMIs.

### 2.5 General procedure for real samples analysis

Three kinds of Chinese baijiu were purchased from the local supermarket, which were used directly for sample testing without pretreatment. For the sample analysis, 10 µL of Baijiu was added to 10 mL of PBS solution (0.1 M, pH 4.0) and the recovery rate was studied by using the standard addition method. Each experiment was repeated three times under the same conditions to obtain the relative standard deviation (RSD).

## 3 Results and discussion

### 3.1 Materials characterization

**FIGURE 2:**
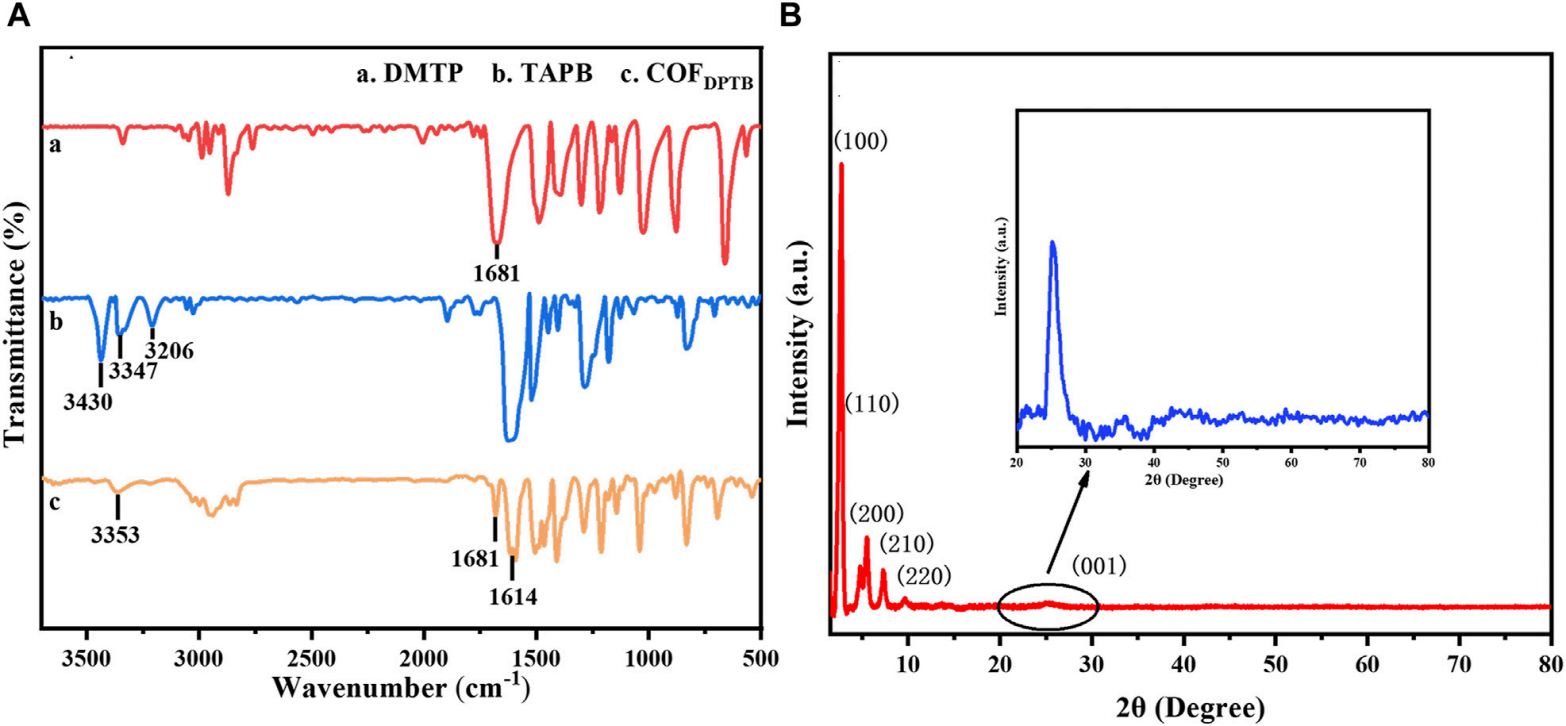
**(A)** FTIR spectra of (a) DMTP, (b) TAPB and (c) COF_DPTB_. **(B)** XRD pattern of COF_DPTB_.

The functional groups of DMTP (curve a), TAPB (curve b) and COF_DPTB_ (curve c) were investigated by FTIR in Figure 2A. In curve a, the characteristic peak at 1,681 cm^−1^ belonged to the C=O stretching vibration of DMTP. As for curve b, the FTIR peaks appeared at 3,430, 3,347, and 3,206 cm^−1^ were originated from the N-H stretching vibration of TAPB. Compared to the two monomers of DMTP and TAPB, the appearance of a new characteristic peak at 1,614 cm^−1^ corresponded to the stretching vibration of C=N (Zhang et al., 2018a), suggesting that the aldehyde group of DMTP formed imine bonds with the amino group of TAPB. Meanwhile, the intensity of the characteristic peaks belonging to DMTP and TAPB decreased significantly, indicating that COF_DPTB_ was generated by the reaction of DMTP and TAPB. XRD was later applied for the phase and structure characterization of the synthesized COF_DPTB_. As shown in Figure 2B, the characteristic diffraction peaks appeared at 2.81°, 4.81°, 5.48°, 7.32°, 9.73°, and 25.39° can be indexed to the plane (100), (110), (200), (210), (220), and (001) of the crystalline COF_DPTB_, respectively, which was consistent with the literature (Zhang et al., 2018b).

**FIGURE 3:**
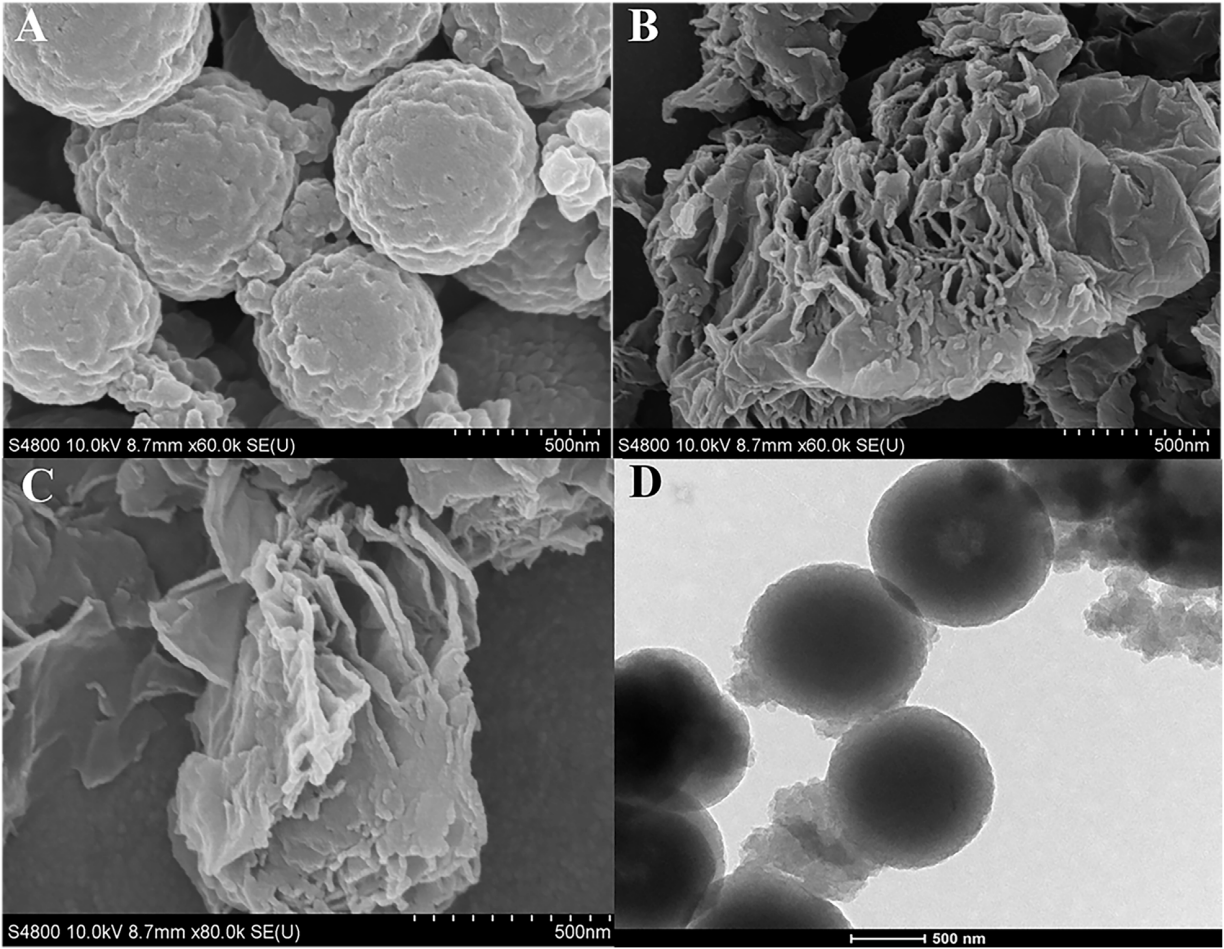
SEM images of **(A)** COF_DPTB_/GCE, **(B)** GR/GCE and **(C)** GR/COF_DPTB_/GCE. **(D)** TEM image of COF_DPTB_.

Next, SEM was used to characterize the surface morphologies of the prepared sensor. In Figure 3A, the SEM images of COF_DPTB_/GCE showed the spherical structure and rough surface, and that of GR/GCE exhibited a lamellar structure in Figure 3B. As shown in Figure 3C, the bottom dark gray rough surface indicated the presence of COF_DPTB_ and the layer structure of GR was clearly, which proved that GR/COF_DPTB_/GCE was successfully obtained. The TEM image of COF_DPTB_ was shown in Figure 3D, which was consistent with its SEM image, proving that it was successful prepared.

**FIGURE 4:**
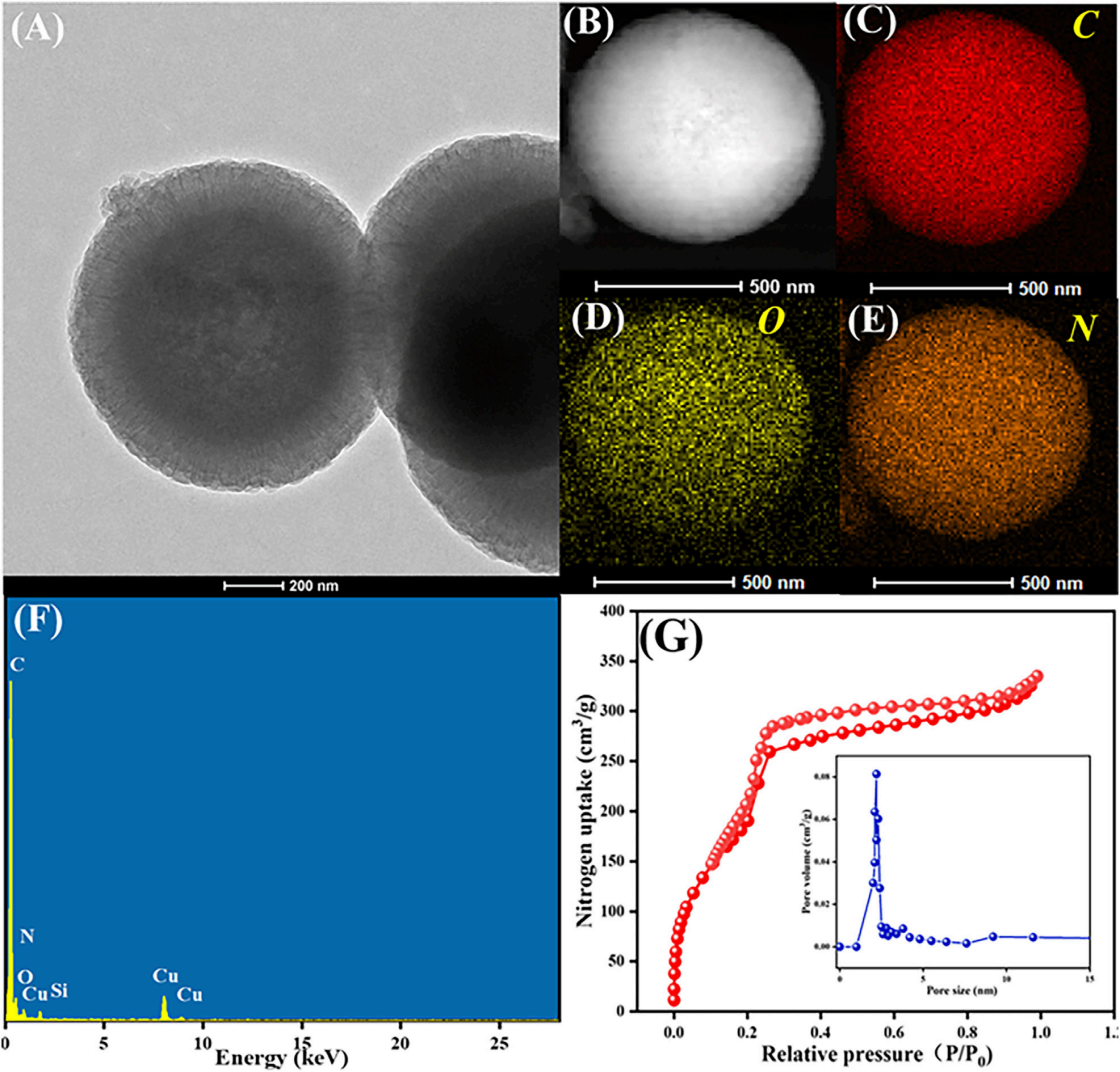
**(A)** HRTEM image, **(B–E)** elemental mapping images, **(F)** EDX spectrum and **(G)** N_2_ adsorption-desorption isotherm (inset: shows the pore size distribution) of COF_DPTB_.

Figure 4 further describes (A) the HRTEM image, (B–E) elemental mapping images, (F) EDX spectrum and (G) N_2_ adsorption-desorption isotherm (inset: shows the pore size distribution) of COF_DPTB_. As Figure 4A shown, the morphology of COF_DPTB_ in HRTEM image was consistent with that of SEM and TEM. In the elemental mapping images (Figure 4B–E), it was observed that the elements of C, O, and N were uniformly distributed. As can be seen from the EDX spectrum in Figure 4F, the content distribution of each element was as follows: C was the highest, N was less, and O was the least. Based on the above investigation, it can be concluded that COF_DPTB_ was successfully synthesized. Figure 4G characterized the porosity of COF_DPTB_ by the N_2_ adsorption-desorption isotherm and the pore size distribution curves (inset), which presented the type IV isotherm with a distinct H3 hysteresis loop, indicating the well-defined mesoporous structure possessed by COF_DPTB_. Its surface area, pore volume and average pore size were measured to be 810.760 m^2^ g^−1^, 0.518 cm^3^ g^−1^, and 2.781 nm, respectively, which greatly improved the effective active sites on the electrode surface.

### 3.2 Electrochemical characterization of the sensor

**FIGURE 5:**
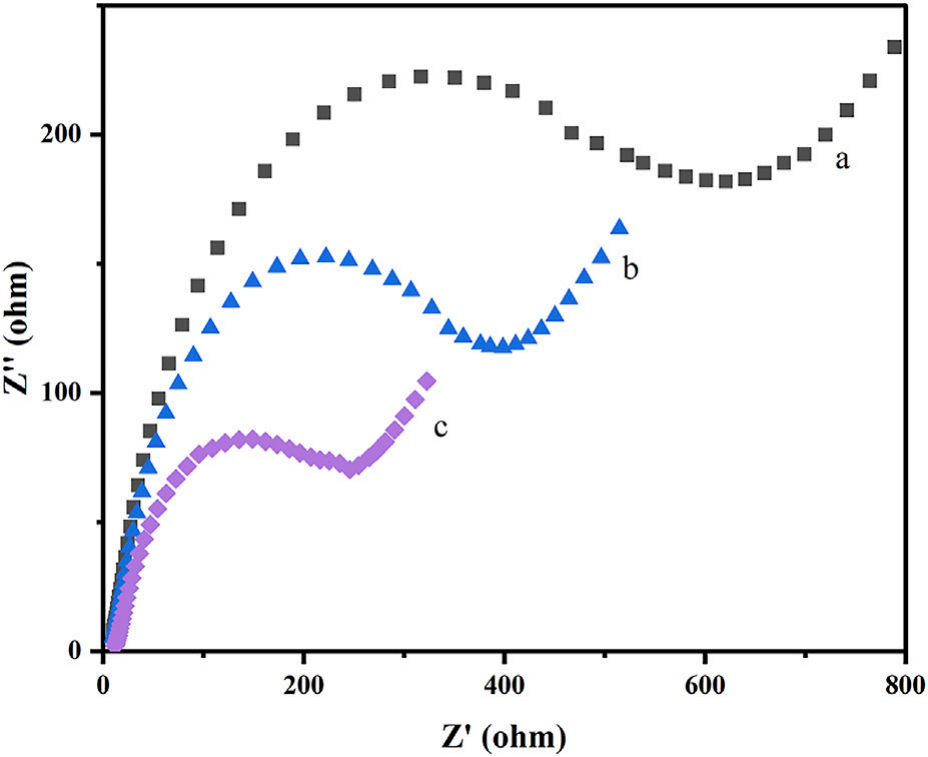
Nyquist plots recorded for (a) GCE, (b) COF_DPTB_/GCE and (c) GR/COF_DPTB_/GCE in 5.0 mM [Fe(CN)_6_]^3–/4–^ with 0.1 M KCl as a supporting electrolyte.

Electrochemical impedance spectroscopy (EIS) was applied to further characterize the behavior of the preparation and assembly process of the prepared sensor. The charge transfer resistances (Rct) of each modification step were estimated according to the semicircle diameters of Nyquist plots in 5.0 mM [Fe(CN)_6_]^3−^/^4−^ probe containing 0.1 M KCl solution. Figure 5 displays the Nyquist plots of the bare GCE (curve a), COF_DPTB_/GCE (curve b), and GR/COF_DPTB_/GCE (curve c). As is shown, the Rct value of the GCE electrode (curve a) was the largest, and it gradually decreased with the deposition of COF_DPTB_ (curve b), indicating that the modifier would promote the surface charge transfer. For GR/COF_DPTB_/GCE (curve c), a significantly reduced semicircle diameter was further observed because the electron transfer rate and mass exchange of the electroactive indicators on the electrode surface were effectively facilitated owing to the synergistically increased specific surface area and high electrical conductivity by COF_DPTB_ combined GR. The results revealed the successful preparation of GR/COF_DPTB_/GCE.

**FIGURE 6:**
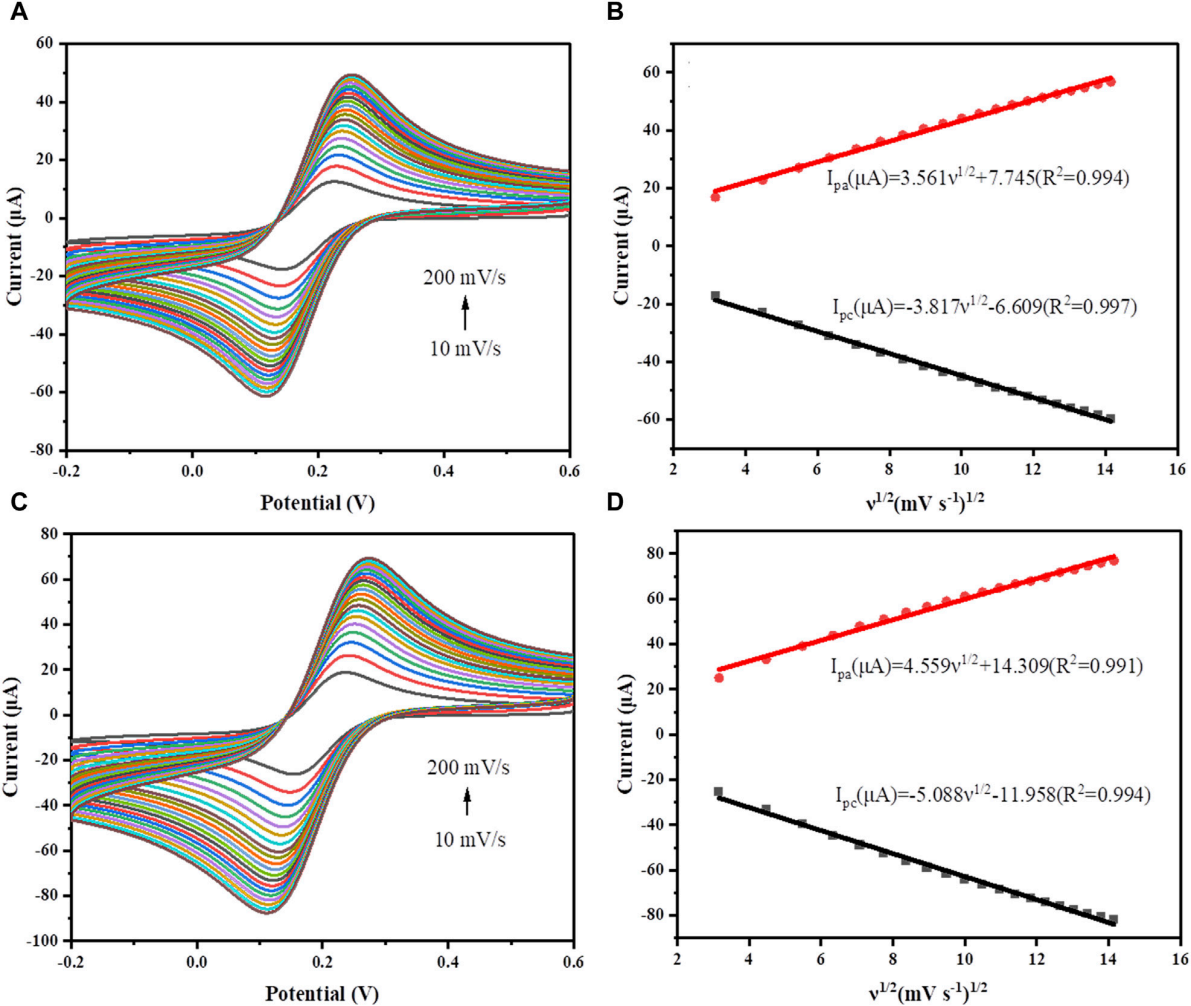
CV curves of **(A)** GCE and **(C)** GR/COF_DPTB_/GCE at different scan rates ranging from 10–200 mV s^−1^ in a mixed solution containing 5.0 mM K_3_Fe(CN)_6_ and 0.1 M KCl. The plots of oxidation and reduction peak currents with the square root of scan rates for **(B)** GCE and **(D)** GR/COF_DPTB_/GCE.

The electroactive surface areas of GCE and GR/COF_DPTB_/GCE were determined by cyclic voltammetry (CV) in 5.0 mM [Fe(CN)_6_]^3−^/^4−^ solution containing 0.1 M KCl (Figure 6). Obviously, both the anodic and cathodic peak currents (*I*_*p*_) were linearly enhanced with the increase of scan rate, and showed a linear dependence on its square root (*v* ^1/2^). The electrochemically active surface areas were calculated by using Randles-Sevcik formula (Dashtian et al., 2019):

$$I_{p}=2.69\times 10^{5}n^{2/3}{AD}^{1/2}C\nu^{1/2}$$

where *I*_*p*_ *, n, A, D, C* and *ν* correspond to the peak current (A), the number of electrons transferred, the electrode’s electrical activity surface areas (cm^2^), the scan rate (V s^–1^), the diffusion coefficient (cm^2^ s^–1^) and the bulk concentration of electrochemically active molecules in this solution (M), respectively. The electroactive areas of GCE and GR/COF_DPTB_/GCE were calculated from the slope of plot of the *I*_*p*_ against *ν* ^1/2^ and found to be 0.046 and 0.068 cm^2^, respectively. It indicated that GR/COF_DPTB_/GCE in [Fe(CN)_6_]^3−^/^4−^ solution showed a fast rate of electron transfer from the modified electrode to the redox probe. The effective surface area of the GR/COF_DPTB_/GCE was 1.48 times higher than that of the GCE, confirming that GR/COF_DPTB_/GCE not only improved the conductivity but also enlarged the active surface area of the modified electrode. It proved that the constructed GR/COF_DPTB_/GCE was a promising candidate for high-performance electrochemical sensor.

**FIGURE 7:**
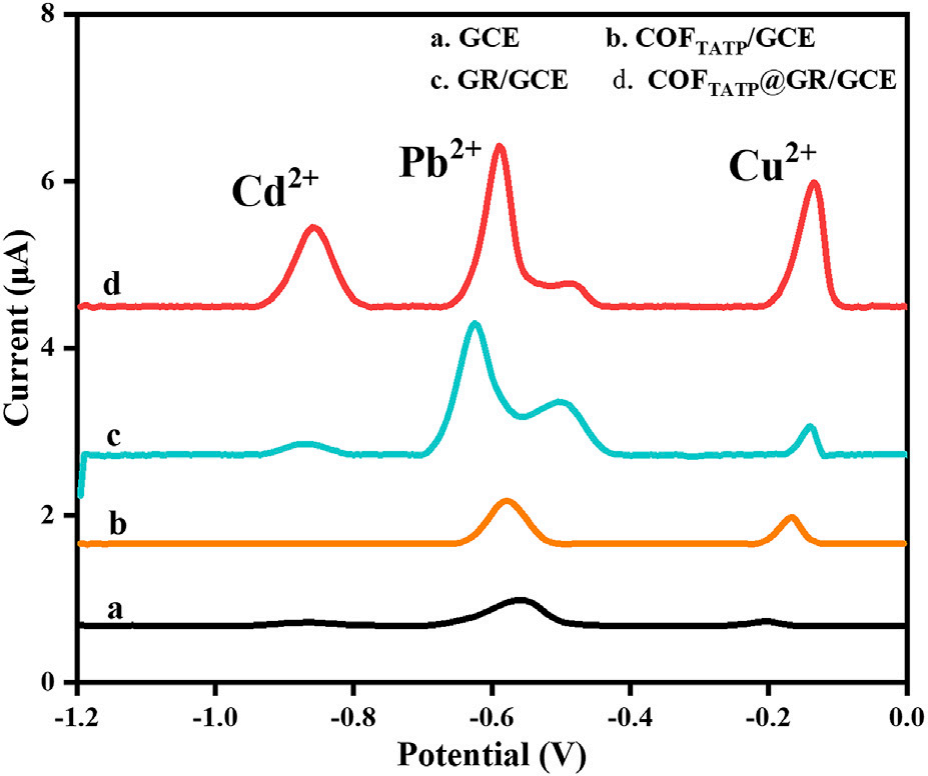
DPASV responses of (a) GCE, (b) COF_DPTB_/GCE, (c) GR/GCE and (d) GR/COF_DPTB_/GCE in 0.1 M PBS (pH 4.0) containing 1.0 μM each of Cd^2+^, Pb^2+^, and Cu^2+^.

To evaluate the detection performance of the proposed electrochemical sensor, the DPASV responses of GCE (curve a), COF_DPTB_/GCE (curve b), GR/GCE (curve c) and GR/COF_DPTB_/GCE (curve d) for 1.0 μM each of Cd^2+^, Pb^2+^, and Cu^2+^ in the N_2_-saturated PBS (0.1 M, pH 4.0) was studied and the results were recorded in Figure 7. As shown, the stripping peak currents of GCE to Cd^2+^, Pb^2+^, and Cu^2+^ were very low (0.090, 0.300, 0.080 μA, respectively). The COF_DPTB_ modified GCE was almost unresponsive to Cd^2+^ and had inconspicuous response to Pb^2+^ and Cu^2+^. The response of GR/GCE to Cd^2+^ and Cu^2+^ was weak (0.146 and 0.442 μA, respectively). It indicated that the GCE, COF_DPTB_/GCE and GR/GCE did not have the ability to detect Cd^2+^, Pb^2+^, and Cu^2+^ at the same time. While for GR/COF_DPTB_/GCE, three anodic stripping peaks of Cd^2+^, Pb^2+^, and Cu^2+^ presented respectively at −0.855 V, −0.590 V and −0.135 V with high response current and large peak-to-peak separation, indicating that there was no mutual interference between the three HMIs. On the one hand, GR can make up for the shortcomings of poor electrical conductivity of COF_DPTB_. On the other hand, the layered graphene and the porous COF_DPTB_ synergistically increased the specific surface area. Moreover, the periodic porous network of COF_DPTB_ enabled the HMIs to be identified and captured selectively, because the N and O atoms on COF_DPTB_ had good selective coordination ability with Cd^2+^, Pb^2+^, and Cu^2+^, which provided a large number of effective binding sites. And the difference in coordination ability with Cd^2+^, Pb^2+^, and Cu^2+^ produced different anodic stripping peaks on the GR/COF_DPTB_/GCE. The above results showed that GR/COF_DPTB_/GCE had the electrochemical analysis ability for the simultaneous detection of Cd^2+^, Pb^2+^, and Cu^2+^. In addition, there was a small shoulder peak near the Pb^2+^ response peak at about −0.48 V, which was attributed to the possible formation of a Pb-Cu alloy (Wang Z. H. et al., 2020).

### 3.3 Analytical parameters optimization

In order to achieve the best electrochemical performance of the developed sensor, DPASV method was used to optimize the relevant experimental parameters, including the dosage ratio of COF_DPTB_ to GR, the pH of PBS solution, the deposition potential and the deposition time.

**FIGURE 8:**
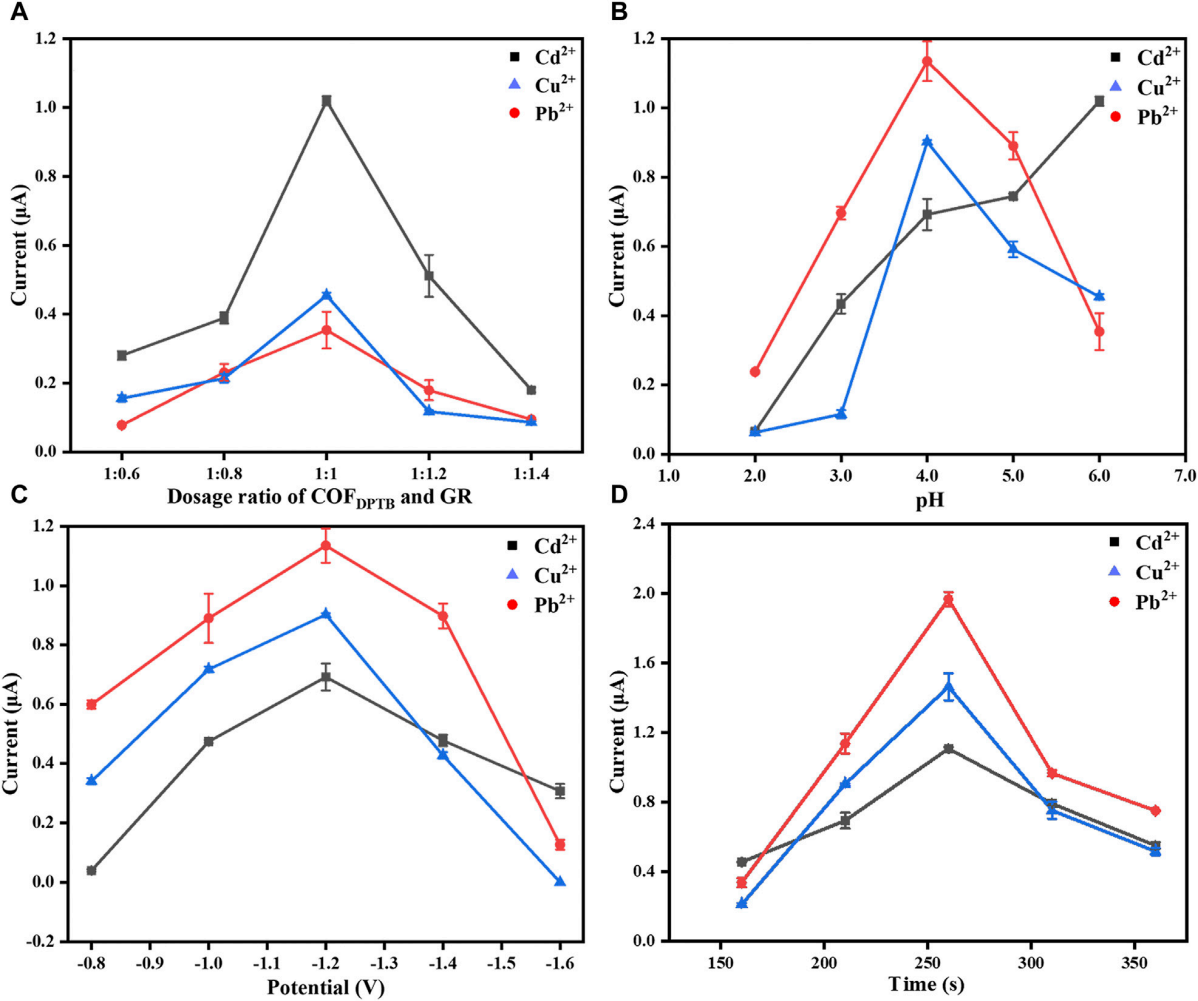
The effect of **(A)** the dosage ratio of COF_DPTB_ to GR, **(B)** the buffer pH, **(C)** the deposition potential, and **(D)** the deposition time on the performance of GR/COF_DPTB_/GCE in 0.1 M PBS containing 1.0 μM each of Cd^2+^, Pb^2+^, and Cu^2+^ under nitrogen atmosphere.

Firstly, the dosage ratio of COF_DPTB_ to GR was optimized. Figure 8A exhibits the influence of the modified dosage ratios of COF_DPTB_ to GR at 1:0.6, 1:0.8, 1:1, 1:1.2, and 1:1.4 on the stripping peak current of the three HMIs. The results showed that the peak currents of the three ions were the highest when the dosage ratio was 1:1. When the ratio was lower than 1:1, the current response was small. It was possibly because of the small content of GR and the low conductivity. And when the ratio was higher than 1:1, the current decreased gradually. It may be because the modified material became thicker, which hindered the mass transfer process of the electrode surface. Therefore, 1:1 was selected as the optimal material modification dosage ratio for the following experiments.

Next, the pH influence of 0.1 M PBS buffer in the range of 2.0–6.0 was studied, as shown in Figure 8B. It can be seen that in this pH range, the peak currents of Pb^2+^ and Cu^2+^ increased first and then decreased, while that of Cd^2+^ increased with the increase of pH. At pH 4.0, the response currents of Pb^2+^ and Cu^2+^ were the highest and Cd^2+^ also had high response current. Under the comprehensive consideration, pH 4.0 was used for the follow-up studies.

The influence of the deposition potential from −0.8 to −1.6 V on the response currents of Cd^2+^, Pb^2+^, and Cu^2+^ was further studied and the results were shown in Figure 8C. From −0.8 to −1.2 V, the stripping signals of Cd^2+^, Pb^2+^, and Cu^2+^ increased gradually because the more negative the deposition potential was, the easier the three ions would be reduced. However, the response currents decreased when it was more negative than −1.2 V, which was due to the increased effect of hydrogen evolution hindering the deposition of the metal alloy being deposited onto the electrode surface (Yu et al., 2018). Thus, −1.2 V was set as the optimal deposition potential for the electrochemical analysis.

Figure 8D displays the DPASV response signals to Cd^2+^, Pb^2+^, and Cu^2+^ in the deposition time range of 160–360 s. As it shown, the current signals increased with the increasing deposition time when it increased from 160 to 260 s. But when it was increased up to 260 s, the DPASV response currents decreased gradually as the deposition time increased, which may be due to the competitive adsorption between Cd^2+^, Pb^2+^, and Cu^2+^, or to the saturated accumulation of the three HMIs mentioned above on the electrode surface that lead to the decreasing of the electron transfer rate at the electrode/solution interface (Isa et al., 2017; Roushani et al., 2017). So, 260 s was chosen as the optimal deposition time.

### 3.4 Calibration curve

**FIGURE 9:**
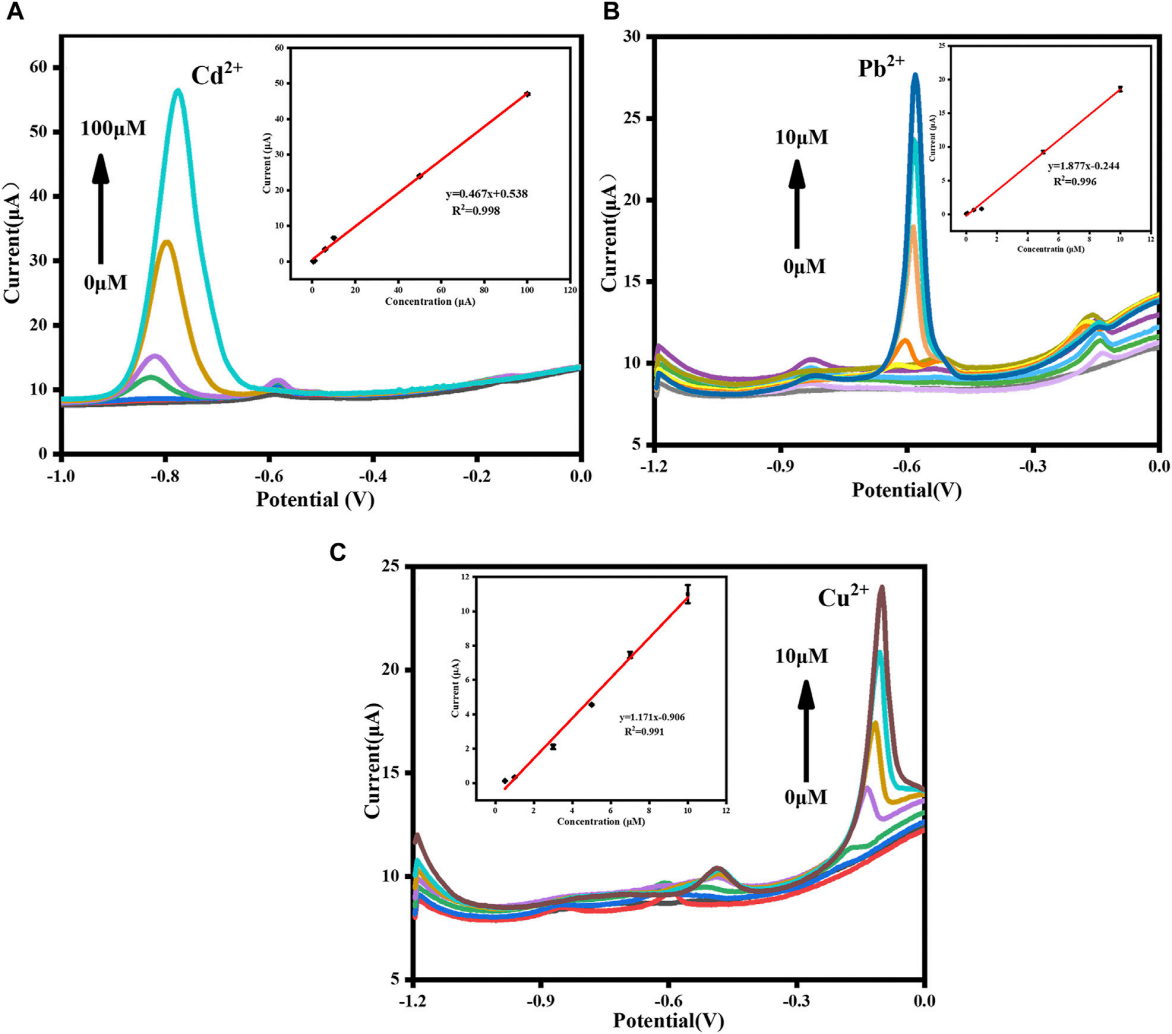
DPASV curves of **(A)** Cd^2+^, **(B)** Pb^2+^ and **(C)** Cu^2+^ detected respectively. The insets are their corresponding calibration curves, respectively.

Under optimal conditions, DPASV responses of Cd^2+^, Pb^2+^, and Cu^2+^ detected by GR/COF_DPTB_/GCE alone and simultaneously were investigated. The individual determination was carried out by increasing the concentration of one target ion and keeping the other two unchanged to study the mutual interference between the three ions. As shown in Figure 9A, the response signals of Cd^2+^ increased accordingly with increasing its amount in the present of 1.0 μM each of Pb^2+^ and Cu^2+^, and the linear regression equation of I (μA) = 0.467 [Cd^2+^ (μM)] + 0.538 (*R* ^2^ = 0.998) was obtained in the range of 0.5–100 μM with the limit of detection (LOD) calculated to be 9.384 nM (S/N = 3). Similarly, as shown in Figure 9B, the analytical curve for Pb^2+^ detection demonstrated a good linearity in the range of 0.01–10 μM and the correlation equation of I (μA) = 1.877 [Pb^2+^ (μM)] − 0.244 (*R* ^2^ = 0.996) was achieved with the LOD calculated to be 4.508 nM (S/N = 3). As for the separate detection of Cu^2+^ shown in Figure 9C, a good linearity in the range of 0.5–10 μM and the correlation equation of I (μA) = 1.171 [Cu^2+^ (μM)] − 0.906 (*R* ^2^ = 0.991) was achieved with the LOD calculated to be 4.987 nM (S/N = 3). The above results verified that no mutual interference existed among the three HMIs.

**FIGURE 10:**
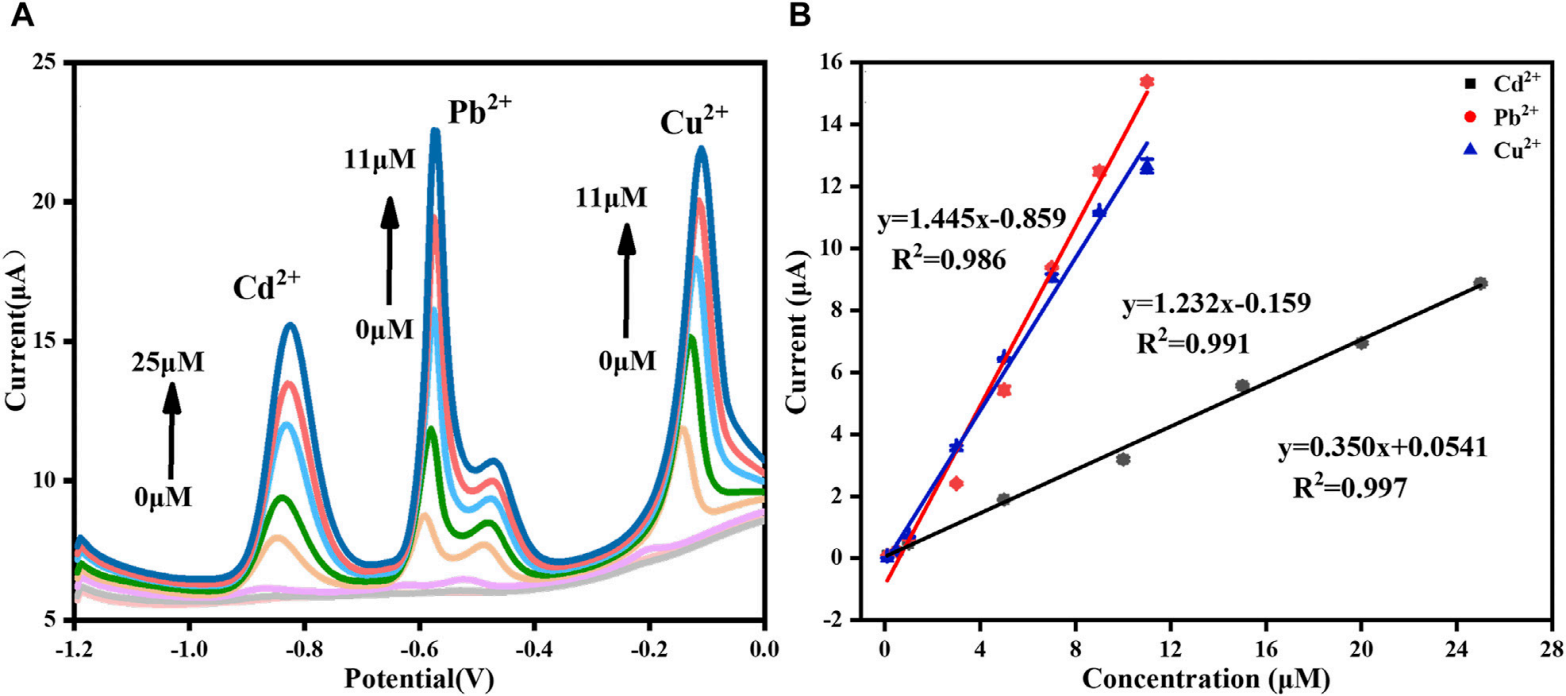
**(A)** DPASV stripping signals of simultaneously detection of Cd^2+^, Pb^2+^, and Cu^2+^ and **(B)** the corresponding respective calibration curves.

In the practical applications, multiple HMIs usually coexist. Therefore, we also studied the linear relationship of the three HMIs detected at the same time, as shown in Figure 10. The results showed that the response current of each ion increased with the increase of its concentration. For Cd^2+^, the linearization equation was I (μA) = 0.350 [Cd^2+^ (μM)] + 0.054 (*R* ^2^ = 0.997) and the corresponding linear range was 0.1–25 μM. For Pb^2+^, the linearization equation was I (μA) = 1.445 [Pb^2+^ (μM)] − 0.859 (*R* ^2^ = 0.986) and the corresponding linear range was 0.1–11 μM. For Cu^2+^, the linearization equation was I (μA) = 1.232 [Cu^2+^ (μM)] − 0.159 (*R* ^2^ = 0.991) and the corresponding linear range was 0.1–11 μM. The LODs of Cd^2+^, Pb^2+^, and Cu^2+^ were 0.011 μM, 8.747 nM and 6.373 nM, respectively (S/N = 3). Compared with the single ion detection, the linear range became narrower, which may be the result of competitive adsorption among the different HMIs (Han et al., 2020). Table 1 summarized the performance comparison of the developed sensor with some other reported electrochemical sensors for the simultaneous detection of Cd^2+^, Pb^2+^, and Cu^2+^. As it shown, the analytical performance of the sensor designed in this study was competitive owing to the widest linear range and the lowest LOD for each HMI. It can be attributed to the large effective surface area and plentiful binding sites provided by the GR/COF_DPTB_ film.

**TABLE 1:** Comparison of the performance of Cd^2+^, Pb^2+^, and Cu^2+^ simultaneously detected by different electrochemical sensors.

Target ions	Method	Modified materials	Linear range (μM)	Limit of detection (μM)	Reference
Cd^2+^	ASV^a^	Ti@TiO_2_	0.6–13.2	0.16	Ma et al. (2015)
Pb^2+^			0.6–13.2	0.14	
Cu^2+^			0.6–13.2	0.1	
Cd^2+^	SWASV^b^	CNT threads	2–6	1	Zhao et al. (2014)
Pb^2+^			0.5–2	0.25	
Cu^2+^			1–3	0.5	
Cd^2+^	DPV^c^	Pd_1.5_/PAC-900^d^	0.5–5.5	0.041	Veerakumar et al. (2016)
Pb^2+^			0.5–8.9	0.05	
Cu^2+^			0.5–5.0	0.066	
Cd^2+^	SWASV	AuNPs@CNFs^e^	0.1–1	0.1	Zhang et al. (2016)
Pb^2+^			0.1–1	0.1	
Cu^2+^			0.1–1	0.1	
Cd^2+^	DPV	AuNP@CDs^f^	0.089–2.402	0.025	Pudza et al. (2020)
Pb^2+^			0.048–1.303	0.02	
Cu^2+^			0.157–4.249	0.220	
Cd^2+^	DPASV	GR/COF_DPTB_	0.1–25	0.011	This work
Pb^2+^			0.1–11	0.008747	
Cu^2+^			0.1–11	0.006373	

^a^ ASV: Anodic stripping voltammetry.

^b^ SWASV: Square wave anodic stripping voltammetry.

^c^ DPV: Differential pulse voltammetry.

^d^ Pd_1.5_/PAC-900: porous activated carbons products loaded with 1.5 wt% Pd by the carbonization treatment at 900°C under N_2_ atmosphere.

^e^ CNFs: carbon nanofibers.

^f^ CDs: carbon dots.

### 3.5 Specificity, reproducibility and stability

It is necessary to study the selectivity of the electrochemical platform because a variety of ions are typically present in Baijiu samples. To examine the selectivity of the proposed sensor, the interference measurements were performed by DPASV under the selected experiment conditions. The applied tolerance limit for the interfering species was the maximum concentration that gave a relative error of ±5% in the presence of 1.0 μM each of Cd^2+^, Pb^2+^, and Cu^2+^, and the results were listed in Table 2. It revealed that 5-fold of inorganic ions (including Ag^+^, Fe^3+^, Zn^2+^, Cr^3+^, Mn^2+^, K^+^, Na^+^, Ca^2+^, Mg^2+^), 1000-fold of small organic molecules (such as methanol, formic acid and lactic acid), and 10^7^-fold of ethanol had no effect on the detection of Cd^2+^, Pb^2+^, and Cu^2+^. It suggested the good specificity of the constructed sensor.

**TABLE 2:** Effects of the interfering ions on the simultaneous detection of Cd^2+^, Pb^2+^, and Cu^2+^ by the developed sensor.

Interfering ions	Concentration	Signal changes (%)		
	(µM)	Cd^2+^	Pb^2+^	Cu^2+^
Ag^+^	5	−2.92	−4.59	−2.07
Fe^3+^	5	2.56	−1.93	1.06
Zn^2+^	5	0.12	−1.30	−4.32
Mn^2+^	5	−4.32	3.83	1.10
Cr^3+^	5	3.00	2.42	−0.47
K^+^, Na^+^, Ca^2+^, Mg^2+^	5	−0.27	−2.85	0.42
Methanol, formic acid and lactic acid	1,000	0.92	0.07	−1.77
Ethanol	10^7^	−4.69	−3.17	1.68

Next, the reproducibility and stability of the fabricated sensor were examined by measuring the DPASV response currents of 0.1 M PBS solution containing 1.0 μM each of Cd^2+^, Pb^2+^, and Cu^2+^ under the optimized working conditions. The repeatability was evaluated by comparing the stripping currents from ten successive measurements on a new prepared GR/COF_DPTB_/GCE and the RSDs were found to be 2.01% for Cd^2+^, 2.56% for Pb^2+^, and 5.29% for Cu^2+^, respectively. The reproducibility was further studied with seven independent GR/COF_DPTB_/GCE sensors, and the RSDs of 4.80% for Cd^2+^, 4.90% for Pb^2+^, and 5.60% for Cu^2+^, respectively were obtained. These results revealed that the developed sensor had good repeatability and reproducibility. When not in use, the prepared sensor was stored at room temperature, and it could respectively retain 91.20%, 96.08%, and 98.18% of Cd^2+^, Pb^2+^, and Cu^2+^ of the original current response after 1 week deposit, indicating good storage stability.

### 3.6 Analysis of real samples

In order to evaluate the practicability of GR/COF_DPTB_/GCE, three different brands of Baijiu purchased from local supermarkets were taken as the samples and the recovery tests were carried out by the standard addition method. The obtained results were listed in Table 3, which showed that the recovery ranges of Cd^2+^, Pb^2+^, and Cu^2+^ were 92.8%–94.2%, 107.8%–109.9% and 92.7%–98.1%, respectively, and RSDs were less than 1.86%, 2.42%, and 4.99%, respectively. The results indicated that the novel sensor had good accuracy and feasibility in the simultaneous assay of Cd^2+^, Pb^2+^, and Cu^2+^ in real samples.

**TABLE 3:** Recovery determination of Cd^2+^, Pb^2+^, and Cu^2+^ in Baijiu samples (*n* = 3).

Target ions	Samples	Original (µM)	Added (µM)	Detected (µM)	Recovery (%)
Cd^2+^	1	Not detected	2.00	1.856 ± 0.035	92.8 ± 1.7
	2	Not detected	2.00	1.875 ± 0.011	93.8 ± 0.6
	3	Not detected	2.00	1.883 ± 0.029	94.2 ± 1.4
Pb^2+^	1	Not detected	1.00	1.078 ± 0.026	107.8 ± 2.6
	2	Not detected	1.00	1.099 ± 0.019	109.9 ± 1.9
	3	Not detected	1.00	1.086 ± 0.014	108.6 ± 1.4
Cu^2+^	1	Not detected	1.00	0.927 ± 0.013	92.7 ± 1.3
	2	Not detected	1.00	0.981 ± 0.025	98.1 ± 2.5
	3	Not detected	1.00	0.954 ± 0.048	95.4 ± 4.8

## 4 Conclusion

In this paper, a novel electrochemical sensor of GR/COF_DPTB_/GCE was prepared by using COF_DPTB_ and GR to simultaneously detect Cd^2+^, Pb^2+^, and Cu^2+^. Under the optimized experimental conditions, the sensor showed the characteristics of low LOD and relatively wide detection range, which was mainly attributed to the large specific surface and abundant binding sites of COF_DPTB_ and the high conductivity of GR. Moreover, the developed sensor exhibited good reproducibility, stability and anti-interference, and has been successfully applied to the simultaneous detection of Cd^2+^, Pb^2+^, and Cu^2+^ in Baijiu. This further expanded the potential application prospect of COFs in the field of food and electrochemical analysis.

## Data Availability

The original contributions presented in the study are included in the article/Supplementary material, further inquiries can be directed to the corresponding authors.
